# Clinical and therapeutic implications of BRAF fusions in histiocytic disorders

**DOI:** 10.1038/s41408-022-00693-7

**Published:** 2022-06-28

**Authors:** Saurabh Zanwar, Jithma P. Abeykoon, Surendra Dasari, Aishwarya Ravindran, Jason R. Young, Aldo A. Acosta-Medina, Karen L. Rech, Jonathan Schwartz, Aaron Mangold, Allison Rosenthal, N. Nora Bennani, Mithun V. Shah, Diana Morlote, Gaurav Goyal, Ronald S. Go

**Affiliations:** 1 Division of Hematology, Mayo Clinic, Rochester, MN USA; 2 Division of Hematopathology, Department of Laboratory Medicine and Pathology, Mayo Clinic, Rochester, MN USA; 3 Department of Radiology, Mayo Clinic, Jacksonville, FL USA; 4 Department of Medicine, Mayo Clinic, Rochester, MN USA; 5 Division of Pediatric Hematology/Oncology, Mayo Clinic, Rochester, MN USA; 6 Department of Dermatology, Mayo Clinic, Scottsdale, AZ USA; 7 Division of Hematology, Mayo Clinic, Scottsdale, AZ USA; 8 Division of Hematopathology, University of Alabama at Birmingham, Birmingham, AL USA; 9 Division of Hematology-Oncology, Division of Hematology-Oncology, University of Alabama at Birmingham, Birmingham, AL USA

**Keywords:** Haematological diseases, Epidemiology

## Dear Editor,

Histiocytic disorders represent a collection of hematologic diseases with varied clinical presentations [1]. The identification of an oncogenic driver has enabled the classification of some of the histiocytic disorders as neoplasms [1]. The activation of the mitogen-activated protein kinase (*MAPK*)-extracellular-signal-regulated kinase (*ERK*) pathway is the hallmark of Erdheim-Chester disease (ECD) and Langerhans cell histiocytosis (LCH) [2]. *BRAF*^V600E^ mutations are identified in 50–60% of patients with LCH and ECD and represent the most conspicuous mechanism for ERK activation [2, 3]. Additionally, one-third of patients with Rosai-Dorfman Disease (RDD) have mutations in the MAPK-ERK pathway [2].

While the MAPK-ERK pathway mutations are ubiquitous in histiocytic disorders, little is known about the prevalence, pathogenic and clinical significance of BRAF fusions. Limited data in the form of case reports suggest that *BRAF* fusions can serve as alternative mechanisms of ERK activation [3, 4, 5], but the implications of BRAF and MEK-inhibitor therapy in histiocytosis harboring *BRAF* fusions are unknown. We conducted this study to examine the frequency, clinical features, and treatment outcomes among patients with histiocytic disorders harboring *BRAF* fusions. We also summarized the published reports of *BRAF* fusions in histiocytic disorders.

After approval by the institutional review board, we screened all new patients with histiocytic disorders seen at our institution between 01/11/2016 and 06/30/2021. All cases with confirmed histopathologic diagnoses of LCH, ECD, RDD, adult xanthogranuloma (AXG), juvenile xanthogranuloma (JXG), histiocytic sarcoma (HS), and Langerhans cell sarcoma (LCS) were analyzed. Only those patients with adequate *BRAF* testing were included in the final study population. Adequate *BRAF* testing was defined as, (i) unequivocally positive for *BRAF*^*V600E*^ immunostain (clone: VE1, Abcam, Cambridge, MA) with or without molecular confirmation or (ii) successful multigene next-generation sequencing with RNA fusion analysis (mostly Tempus^®^ or FoundationOne^®^; required if VE1 immunostain was equivocal or negative). We also performed an extensive literature review for reports of *BRAF* fusions among histiocytic disorders through the PubMed search engine by using keywords: “*BRAF* fusion”, “histiocytosis”, “histiocytic disorders”, “Langerhans cell histiocytosis”, “Erdheim-Chester disease”, “Rosai-Dorfman disease”, “xanthogranuloma”, “Langerhans cell sarcoma”, and “histiocytic sarcoma”. Immunostaining for phospho-ERK (p-ERK, clone: D13.14.4E, Cell Signaling, Danvers, MA) was performed on formalin-fixed paraffin-embedded tissue sections using standard immunohistochemical methods on automated staining platforms and reviewed by two pathologists (K.L.R. and A.R.). Response assessment was defined based on the Consensus recommendations.

One hundred and twenty-six patients with a diagnosis of histiocytic disorder and adequate *BRAF* testing were identified. *BRAF* fusions were detected in seven (6%) patients. The frequency of *BRAF* fusions according to disease subtypes in our cohort was as follows: AXG/JXG (4/7 [57%]), ECD (2/46 [4%]), LCH (1/41 [2%]), RDD (0/23 [0%]), and HS/LCS (0/9 [0%]). The median age at diagnosis for patients with *BRAF* fusion cases was 34 years (range, 7–81 years) and 5 (71%) were females. We also identified 16 cases of histiocytosis with *BRAF* fusions reported in the literature. The clinical and molecular characteristics from our cohort as well as the previous reports are shown in Table 1. In the combined cohort of 23 patients, the median age at diagnosis was 19 years (range, 0.5–81 years) and 60% were females. The distribution of *BRAF* fusions by disease subtypes was as follows: AXG/JXG (10/23, 43%), LCH (7, 30%), ECD (3, 13%), non-LCH not otherwise specified (2, 9%), and HS/LCS (1, 4%). Most of the patients (13, 56%) had a single-system disease. The skin was the most common site of involvement (11, 50%) followed by bone (7, 32%), brain (5, 23%), and lung (4, 18%).

**Table 1:** Summary of clinical characteristics of patients with BRAF fusions in patients with histiocytic disorders.

Cohort	Age (yrs)/ sex	Type	BRAF fusion	Organ involvement	Frontline therapy	Response
MC-1	27/F	ECD	*UBTD2-BRAF*	Brain, bone	Cobimetinib	PR (sustained 12 months)
MC-2	^32/F^	ECD	*RNF11-BRAF*	Bone, kidney, heart, lung, sinus	Cobimetinib	PR (sustained at 8 months)
MC-3	55/F	LCH	*LMTK2-BRAF*	Bone	Radiation + zoledronic acid	PR
MC-4	81/M	AXG	*AGAP3-BRAF*	Skin, multicentric	Observation	-
MC-5	34/F	AXG	*ARRB1-BRAF*	Skin, multicentric	Observation	-
MC-6	60/F	AXG	*UBR2-BRAF*	Lung, sclera, skin (disseminated)	Observation	-
MC-7	7/M	JXG	*FNBP1-BRAF*	Spinal cord	Surgery + clofarabine	CR
LR-1^2^	4/M	LCH	*BICD2-BRAF*	Bone	NA	-
LR -2^2^	37/F	LCH	*CSF2RA-BRAF*	Thyroid, node, salivary gland	NA	-
LR -3^2^	57/M	LCH	*PACSIN2-BRAF*	Node, oral mucosa	NA	-
LR-4^2^	29/F	LCH	*SPPL2A-BRAF*	Skin	NA	-
LR -5^2^	1/M	JXG	*RNF11-BRAF*	Skin	NA	-
LR -6^2^	0.5/M	JXG	*MS4A6A-BRAF*	Skin	NA	-
LR -7^2^	14/M	JXG	*BICD2-BRAF*	Brain	NA	-
LR -8^2^	12/F	JXG	*BICD2-BRAF*	Skin (disseminated), bone, node, lung	NA	-
LR -9^4^	16/F	HS/LCS	*MTAP-BRAF*	Subcutaneous	Surgery	CR
LR -10^4^	12/F	JXG	*MS4A6A-BRAF*	Lung, node, skin (disseminated)	Clofarabine	PR
LR -11^3^	6/M	LCH	*PACSIN2-BRAF*	Bone, skin	Prednisone/vinblastine	SD
LR -12^6^	NA	ECD	*PICALM-BRAF*	Bone, brain	Vinblastine/etoposide/interferon	Progression
LR -13^10^	15/NA	LCH	*FAM73A-BRAF*	Single system, single lesion disease (details not available)	NA	-
LR-14^14^	14/NA	Non-LCH	*RNF11-BRAF*	Brain	NA	-
LR-15^14^	38/F	Non-LCH	*CLIP2-BRAF*	Retroperitoneum	NA	-
LR-16^15^	22/F	AXG	*GAB2-BRAF*	Skin, brain (pituitary)	Prednisone	PR

*CR* complete response, *F* female, *LR* cases from literature review, *M* male, *MC* Mayo clinic, *NA* not available, *PR* partial response, *SD* stable disease.

**Fig. 1:**
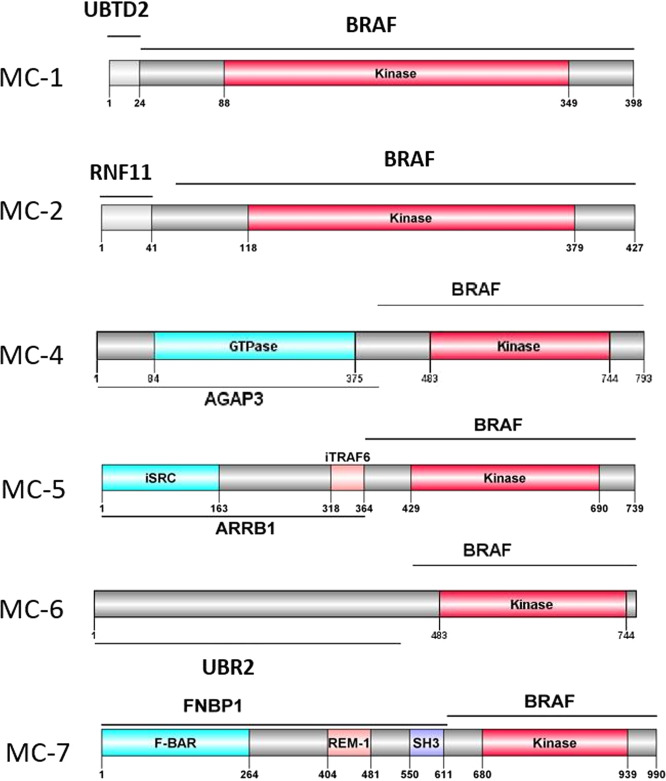
Locations of *BRAF* fusion. Patients in the Mayo Clinic cohort with *BRAF* fusions were noted to have preserved kinase domain regions.

We identified 17 different *BRAF* fusions with several being recurrent (*RNF11-BRAF* in 3, *BICD2-BRAF* in 3, *PACSIN2-BRAF* in 2, and *MS4A6A-BRAF* in 2; Table 1). In the Mayo Clinic cohort, the data on the breakpoints of the *BRAF* fusions were available for six patients. All six of these *BRAF* fusions had intact kinase domain regions, Fig. 1. Three patients (MC-4, MC-6, and MC-7) had adequate tissue available for p-ERK immunohistochemistry and demonstrated moderate to strong nuclear and cytoplasmic (2–3+) p-ERK expression (Supplementary Fig. 1).

In our cohort, two patients with ECD underwent treatment with a MEK inhibitor (cobimetinib). The first patient (MC-1) harbored *UBTD2-BRAF* fusion and completed 12 cycles (12 months) of cobimetinib resulting in partial response (PR) in the lesions of brain parenchyma and tibia, with a first response within 2 months after initiation of cobimetinib (Supplementary Fig. 2). She also received intra-arterial melphalan for the residual brain parenchymal lesion, resulting in further tumor shrinkage. She continued to be in a sustained PR at the last follow-up 2.5 years from diagnosis. The second patient (MC-2) who underwent cobimetinib treatment had *RNF11-BRAF* fusion and achieved PR in the perirenal soft tissue, vertebral lytic lesion, along with a resolution of the bilateral pleural effusions at 2 months. She developed intolerable adverse effects (fatigue, rash, diarrhea, fever, nausea, and vomiting) after two cycles of cobimetinib resulting in treatment discontinuation but remained in a sustained PR 6 months after drug discontinuation. From a literature review, one patient (LR-12) was treated with cobimetinib as a second-line treatment and achieved a complete response [6].

While point mutations in *BRAF* are well-described in ECD and LCH, data on *BRAF* fusions are limited. Our series represents the largest study to date focusing on patients with histiocytosis and *BRAF* fusions. The presence of a *BRAF* fusion was uncommon in our overall cohort (~5%), but quite common in AXG/JXG subgroup (>30%). *BRAF* fusions are also an uncommon occurrence in most other neoplasms. A previous report utilizing comprehensive genomic profiling of solid tumors identified the presence of a *BRAF* fusion in 55 out of 20,573 (0.3%) patients, most notably in melanomas and pilocytic astrocytomas [7]. Interestingly, certain neoplasms with ectodermal origins have a high proportion of kinase fusions including *BRAF*. The spitzoid tumors/spitzoid melanomas harbor kinase gene fusions (including *ALK*, *BRAF*, *NTRK*, and *ROS1*) in up to 50% of patients [8] while pilocytic astrocytomas demonstrate *BRAF* fusions in 25–40% of the cases [9]. Apart from the common ectodermal origin, other associations between these tumors are limited and it is difficult to postulate with confidence as to why the AXG/JXG patients are enriched in *BRAF* fusions. On comparing the fusion partners of *BRAF* between our cohort and previous reports in solid tumors, all except the *AGAP3-BRAF* were novel [7]. Similar to our findings, previous reports of *BRAF* fusions in melanocytic tumors and histiocytic disorders have also demonstrated intact *BRAF* kinase domains [4, 10].

Increased ERK phosphorylation has been demonstrated in melanoma cell lines with induced *BRAF* fusions further suggesting the functional potential of these fusions [10, 11]. In our cohort of seven patients, three had adequate tissues for p-ERK Immunohistochemistry. All three patients expressed p-ERK (2+ to 3+), further strengthening the hypothesis that these *BRAF* fusions cause downstream *MAPK-ERK* activation. Similarly, we have recently demonstrated in a case of *CSF1R*-mutated ECD that mutation outside of the MAPK pathway was associated with negative p-ERK immunohistochemistry, with no response to MEK-inhibitor therapy [12]. While the evidence of functionality of *BRAF* fusion is defined, there are limited data on the role of targeted therapy in patients harboring these fusions. The response to MEK inhibition in our study suggests that this may be an effective treatment strategy for patients harboring *BRAF*-fusions like other *MAPK-ERK-*activated histiocytosis. Interestingly, *RAF* inhibition in patients with *BRAF* fusions may not be an effective strategy. A prior study of melanocytic tumors cells lines with *BRAF* fusions demonstrated a paradoxical RAS-independent MAPK activation upon treatment with first- and second-generation *RAF* inhibitors and this was attributed to the fusion partners for *BRAF* in these cell lines - *FKBP15-BRAF* and *SKAP2-BRAF* [13]. Additionally, the *RNF11-BRAF* fusion is noted to sensitize murine pro-B cell Ba/F3 cells to MEK inhibition, but not RAF inhibition by vemurafenib [14, 15]. It is unclear if a concomitant BRAF and MEK inhibition would lead to better outcomes in these patients and further studies are needed to determine the role of combination therapy.

In summary, we report a robust collation of cases with histiocytic disorders harboring *BRAF* fusions from our institution and the existing literature. *BRAF* fusions are enriched among patients with xanthogranuloma, both in pediatric as well as adult populations. Most cases had preserved kinase domains of the *BRAF* gene, representing an alternate mechanism of ERK activation and potentially providing a therapeutic opportunity using *MEK* inhibitors.

## Supplementary information

Supplementary Material
