# Supplementary material for: Clinical and therapeutic implications of BRAF fusions in histiocytic disorders
Source: Blood Cancer J. 2022 Jun 28;12(6):97. doi: 10.1038/s41408-022-00693-7 (PMC9240055; doi:10.1038/s41408-022-00693-7)
Supplement: Supplementary file 1 — Supplementary Material [file 41408_2022_693_MOESM1_ESM.docx]

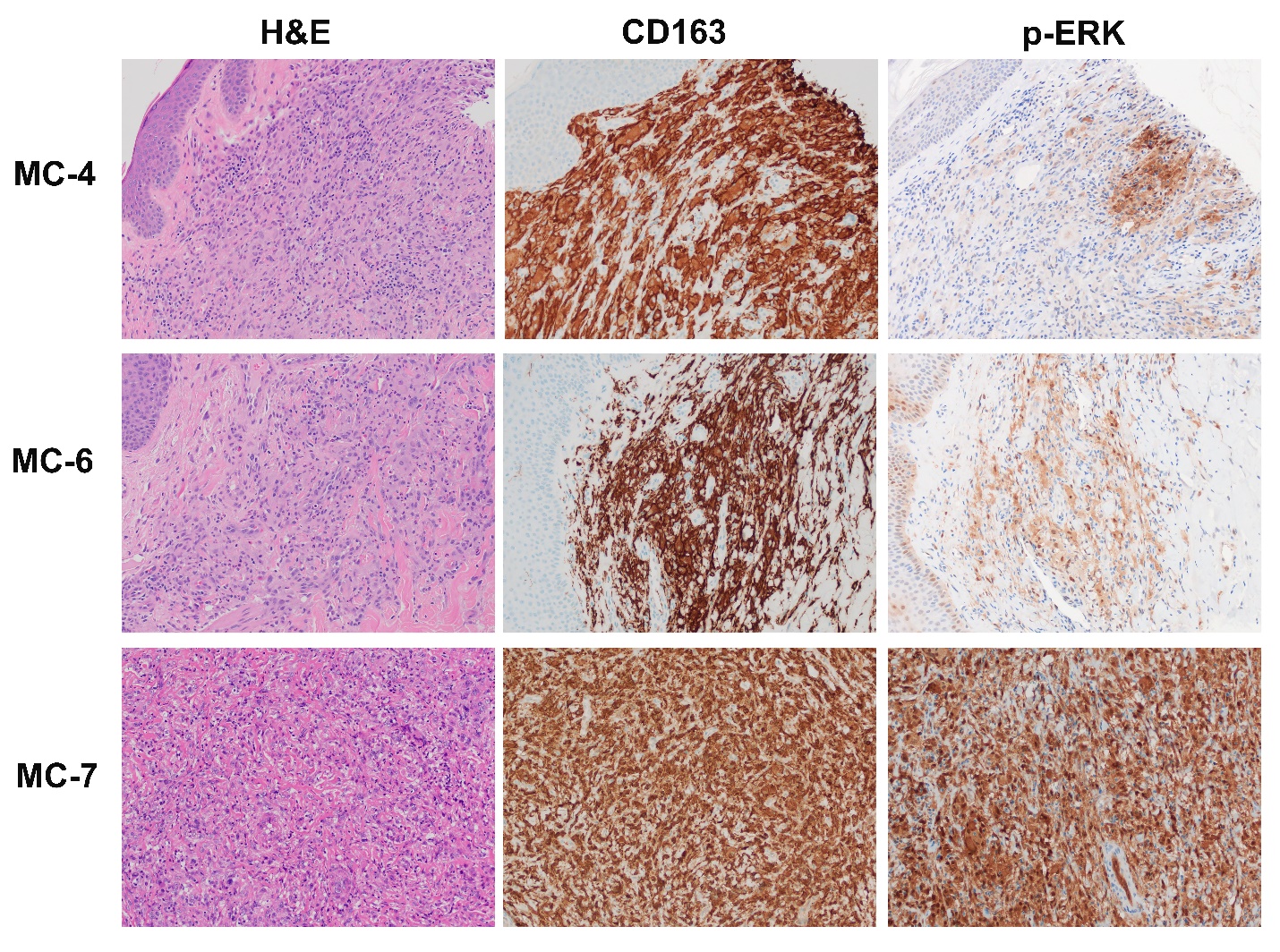


**Supplementary figure 1:** Light microscopy showing hematoxylin and eosin (H&E) stained tissue sections involved by CD163-positive lesional histiocytes with p-ERK expression (nuclear and cytoplasmic staining): MC-4 (skin): focal strong (3+); MC-6 (skin): moderate (2+); MC-7(spinal cord): strong (3+)


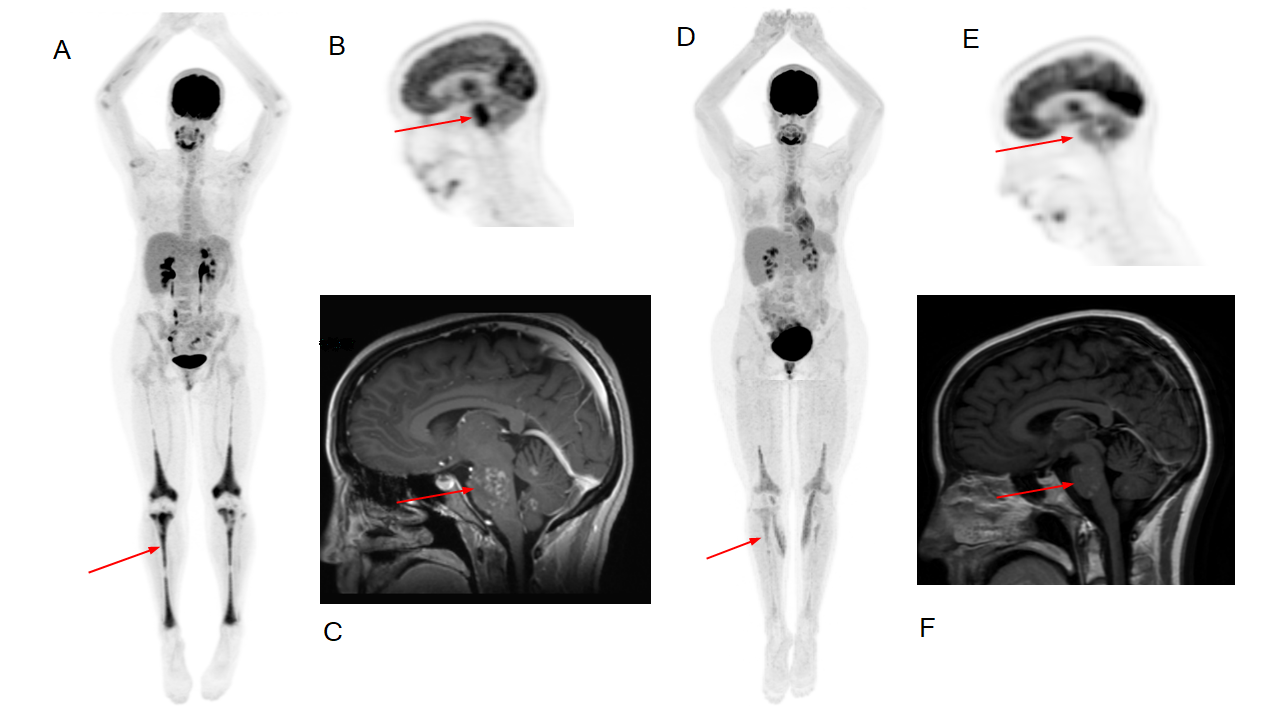


**Supplementary Figure 2.** Response assessment for a patient with a *UBTD2-BRAF* treated with a cobimetinib. F-18 flurodeoxyglucose positron emission tomography demonstrates a partial response with SUV_max_ in the femur decreasing from 6.4 (A) to 2.6 (D) and SUV_max_ in the pons decreasing from 11.3 (B) to 8.4 (E). Corresponding contrast enhanced MRI images demonstrate decreased mass effect and enhancement of the pons after treatment with cobimetinib for 12 months (C and F).
